# Glucose Sensing for Diabetes Monitoring: Recent Developments

**DOI:** 10.3390/s17081866

**Published:** 2017-08-12

**Authors:** Danielle Bruen, Colm Delaney, Larisa Florea, Dermot Diamond

**Affiliations:** Insight Centre for Data Analytics, National Centre for Sensor Research, School of Chemical Sciences, Dublin City University, Dublin 9, Ireland; danielle.bruen2@mail.dcu.ie (D.B.); colm.delaney@dcu.ie (C.D.); dermot.diamond@dcu.ie (D.D.)

**Keywords:** diabetes, glucose, sensing, non-invasive, ocular fluid, sweat

## Abstract

This review highlights recent advances towards non-invasive and continuous glucose monitoring devices, with a particular focus placed on monitoring glucose concentrations in alternative physiological fluids to blood.

## 1. Introduction

*Diabetes mellitus* is an incurable disease [1] resulting from an insufficiency of insulin in the body [2], causing elevated blood-glucose levels, known as hyperglycaemia, or reduced glucose concentrations, known as hypoglycaemia [3,4]. Insulin is a hormone which is synthesised and secreted from the pancreas to mediate metabolic reactions involving glucose [5,6]. In doing so, it initiates glucose uptake by cell types in the body, hence reducing glucose concentrations in blood [2,7]. Diabetes has been associated with many medical conditions, including coeliac disease, cystic fibrosis, tuberculosis and heart disease. Such complications can result in retinopathy leading to blindness, nephropathy giving rise to renal failure, peripheral nerve damage with increasing risks of extremity (foot) ulcers, amputation, cardiovascular diseases or even cancer [2,4,6]. Diabetes has been described as a “silent epidemic” in a review by Nwaneri detailing the origin of the disease [8] and since its discovery, great efforts have been made to achieve efficient diagnosis, monitoring and treatment.

A plethora of biosensors have been developed to provide diagnostic information regarding a patient’s health status. Many different types of sensors have been investigated, and a 2010 review by Toghill and Compton provides a great insight into enzymatic and non-enzymatic electrochemical glucose sensing approaches studied over the past decade [9]. Spectroscopic methods for non-invasive glucose detection have also been growing in popularity, with Raman and infrared spectroscopy being the focus of particular attention [10,11,12]. For applications of clinically relevant biosensors the reader is directed to excellent reviews by Corrie et al. [13] and Yoo et al. [14]. However, the real challenge that remains is the creation of biosensors for daily use by patients in personalised monitoring [14]. Several reviews focusing on sensor integration into wearable platforms have been published recently [15,16,17], therefore, this review focuses solely on recent advances pertaining to biological fluids other than blood, such as interstitial fluid, sweat, breath, saliva and ocular fluid, and focusing particularly on non-invasive approaches which may enable continuous glucose monitoring for diabetics.

## 2. Glucose Monitoring

### 2.1. Glucose Monitoring Methods in Blood

As a preventative treatment or cure for diabetes is yet to be developed, managing the life-impeding conditions of this disease is currently the most successful means for its control. Monitoring glucose levels in blood, as a disease marker, has proven to prolong life expectancy by enabling diabetics to manage episodes of hypo- or hyperglycaemia, hence providing better control over their condition and preventing some of the debilitating side effects [18,19]. In addition, glucose monitoring can be used to optimise patient treatment strategies, and provide an insight into the effect of medications, exercise and diet on the patient [13]. Although blood-glucose monitoring is the gold-standard medium for glucose sampling, measurements carried out in this fluid are invasive [16,18]. Blood-glucose concentrations are typically in the range of 4.9–6.9 mM for healthy patients, increasing to up to 40 mM in diabetics after glucose intake [2,18,19,20,21].

Clark and Lyons at the Children’s Hospital in Cincinnati proposed in 1962 the first-generation of glucose biosensors [22]. These sensors were initially based on an electrochemical approach, which used the enzyme glucose oxidase (GOx) [13]. Electrochemical sensors were chosen for blood-glucose measurements due to their high sensitivity, on the order of μM to mM, good reproducibility and ease of fabrication at relatively low cost [18]. GOx was employed as the enzymatic basis for the sensor, owed to its high selectivity for glucose. Less common enzymes, such as hexokinase and glucose-1-dehydrogenase were also used for glucose measurements [23,24], but GOx can tolerate extreme changes in pH, temperature and ionic strength in comparison with other enzymes. Withstanding these conditions can be important during any manufacturing processes, making it a prime candidate for glucose monitoring devices [25,26].

**Scheme 1:**
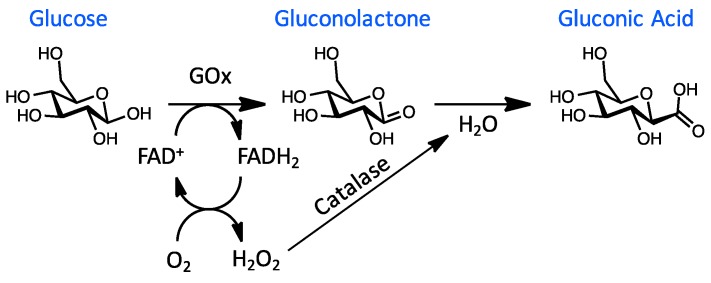
Conversion of glucose to gluconic acid using glucose oxidase. Adapted from [28].

GOx catalyses the oxidation of glucose to gluconolactone in the presence of oxygen, while producing hydrogen peroxide (H_2_O_2_) and water as by-products (Scheme 1) [13]. Gluconolactone further undergoes a reaction with water to produce the carboxylic acid product, gluconic acid. GOx requires a redox cofactor to carry out this oxidation process, where flavin adenine dinucleotide (FAD^+^) is employed. FAD^+^ is an electron acceptor which becomes reduced to FADH_2_ during the redox reaction [27]. Subsequent reaction with oxygen to produce H_2_O_2_ regenerates the FAD^+^ cofactor. This reaction occurs at the anode, where the number of transferred electrons can be correlated to the amount of H_2_O_2_ produced and hence the concentration of glucose [27].

In the sensor design presented by Clark and Lyons, indirect quantification of glucose concentrations was achieved by placing a thin layer of the GOx enzyme on a platinum electrode via a semipermeable dialysis membrane. This sensor measured the decrease in oxygen concentration and the liberation of hydrogen peroxide, which was proportional to the glucose concentration [22]. The main obstacle to overcome with this approach was the interference of other electroactive species present in blood, such as ascorbic acid and urea [13,29]. This approach was further developed in 1975, when the first successful commercial sensor based on GOx was made available [13]. This sensor directly measured glucose concentrations by amperometric detection of hydrogen peroxide. The electrochemical signal required a high operating potential and due to the expensive nature of the platinum electrode used, the use of this device was strictly confined to clinical settings [13]. This led to the second generation of glucose-biosensors in the 1980s [29].

In the design of first generation sensors, oxygen was employed as the electron-acceptor, which can result in errors from variations in oxygen tension and limitations, known as the oxygen deficit [30]. This deficit is caused by oxygen concentrations being one order of magnitude lower than measured glucose concentrations. In order to overcome these challenges, oxygen was replaced with a synthetic electron redox mediator in second generation sensors [30]. The evolution of this sensing approach also led to the development of disposable enzyme electrode strips, which were accompanied by a pocket-size blood-glucose meter [31,32]. Each strip housed miniaturised screen-printed working and reference electrodes, where the working electrode was coated with the required sensing components; glucose oxidase, an electron-shuttle redox mediator, stabilizer and linking agent. These revolutionary second-generation glucose sensors directly resulted in the advent of self-monitored glucose management, known as the “finger-pricking” approach.

**Figure 1:**
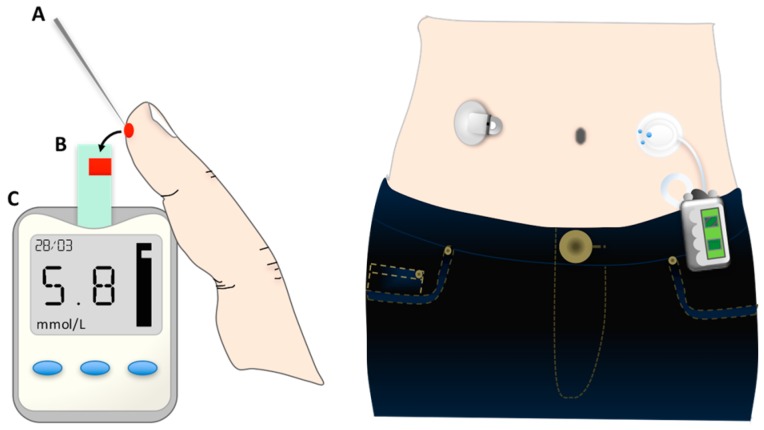
Finger pricking device (**left**). A. Lancet needle; B. Blood sample on test-strip; C. Glucose meter displaying glucose concentration in mmol/L. Continuous glucose monitor (**right**). Adapted from [46,47].

Currently, the most widely used self-monitoring method involves this ‘finger-pricking’ approach, is enzymatic-based, and involves sampling blood from a finger via pricking, to be analysed by in vitro methods using test strips and a glucometer (Figure 1) [7,21,33,34,35,36,37,38,39,40,41,42]. The effectiveness of this method relies on strict compliance, which can be negatively influenced by time constraints, pain, and inconvenience [43]. It is also not a continuous monitoring approach [21,36,37,39,44] and needs to be carried out at multiple intervals throughout the day to help manage elevated glucose levels [18,20,21,37], especially after meals [41], exercise [20,41] and dosing of insulin medication [20,41,43,45]. Moreover, a non-continuous method such as this can overlook periods of hyper- or hypoglycaemia which occur outside of the sampling window [43]. Recent developments in implantable sensors, on the other hand, can be used to incorporate insulin pumps, which allow for instant insulin administration [20,35,42,43,45].

In the early 1970s, Albisser et al. and Shichiri et al. first introduced in vivo continuous glucose monitoring using an artificial pancreas [48,49]. The artificial pancreas design was based on continuous glucose monitoring, where the device would remove blood from the body to an external benchtop analyser that was connected to an insulin pump. As this suggests, the device was not implanted and therefore not portable, although it was named the ‘artificial pancreas’. This led to the development of a third generation of glucose biosensor, which was subcutaneously implanted (Figure 1). Although the device could analyse glucose concentrations in blood using GOx, this was considered an invasive method [50]. It wasn’t until the late 1990s that the first commercially available personalised in vivo glucose monitor was launched by Medtronic Minimed Inc. (Sylmar, CA, USA) [29]. Unfortunately, the device could not provide real-time information, with data being accessed by a physician every 3 days [13]. Although implantable glucose monitoring systems offer regular glucose level readings, this approach isn’t recommended for all diabetics, due to its invasive nature [18] and some continuous glucose monitoring methods have been reported to show inaccuracies of up to 21% [51]. These inaccuracies are often attributed to sensor drift, caused by changes in the catalytic performance of the enzyme. This requires the device to be periodically recalibrated via the finger-pricking method [52]. Despite current commercially available glucometers, such as the Freestyle-Navigator by Abbott (Abbott Park, IL, USA), providing real-time measurements every 1–5 min, the longest working model without calibration is approximately two weeks. Consequently, there is high consumer demand for a continuous glucose monitoring system which can quantify glucose concentrations without frequent calibration. Although blood remains the most studied body fluid for such measurements, other more accessible biological fluids such as interstitial fluid, ocular fluid, sweat, breath, saliva or urine have been investigated as alternative sample media for non-invasive continuous monitoring (Table 1) [16,17,18,19]. It is likely that the development of a device for glucose sensing with a working model of more than two weeks may target one of these more accessible fluids.

### 2.2. Monitoring Glucose in Alternative Physiological Fluids

#### 2.2.1. Interstitial Fluid

Interstitial fluid is the extracellular fluid which surrounds tissue cells. It has significant potential for medical diagnostics as it possesses a similar composition of a number of clinically important biomarkers to blood [14,15]. Blood and the surrounding vascularised tissue readily exchange biological analytes and small molecules by diffusion with the interstitial fluid [14]. As a result, the interstitial fluid can offer valuable information about a patient’s health and has been used for minimally invasive determination of inherited metabolic diseases, organ failure or drug efficacy. Consequently, substantial efforts have focused on non-invasive glucose sensing in this physiological fluid to provide information regarding a patient’s glycaemic state.

Methods for monitoring glucose via the skin have become very popular in recent years, where these approaches have been developed to counteract the challenges associated with patient compliance and invasive monitoring [15]. Some of these approaches include sensing by optical detection such as light absorption or fluorescence detection, ultrasound or sonophoresis, polarimetry, heat or thermal emission, electromagnetic techniques, photoacoustic detection, Raman or bioimpedance spectroscopy, electrochemical methods and reverse iontophoresis-based electrochemical sensing, among others (Table 2) [15,19,61,62,63]. A limitation of reverse iontophoresis is that typically stable measurements can only be reliably recorded for a period of 24 h before calibration of the device is required. This is thought to result from the initiation of the skin healing process as interstitial fluid sampling is achieved by breaching the skin barrier [62]. The GlucoWatch was developed as a wearable device which was initially brought to market for non-invasive continuous monitoring of glucose [1,20,33,35,45]. This technique used reverse iontophoresis to extract interstitial fluid through the skin, and measure glucose levels [1,33] in a pH range of pH 7.2–7.4 [14]. Although the GlucoWatch was a considerable advancement towards non-invasive and continuous glucose monitoring, the approach was hampered by the need for periodic recalibration by the pricking method [13,35,64], thereby resulting in an increase in costs for testing equipment and patient care [45]. Other drawbacks included long warm up times, sweating and skin rash with irritation [35], which subsequently resulted in this product’s removal from the market in 2008 [13,15,61].

Sode et al. have also developed a self-powered implantable continuous monitoring device called the BioRadioTransmitter for use in an artificial pancreas [68]. In this instance, the device is composed of a capacitor, radio transmitter and receiver. In the presence of glucose, the capacitor of the BioRadioTransmitter device discharges a radio signal, which is received and amplified by the radio receiver. The change in transmission frequency is then related to the glucose concentration [68].

**Figure 2:**
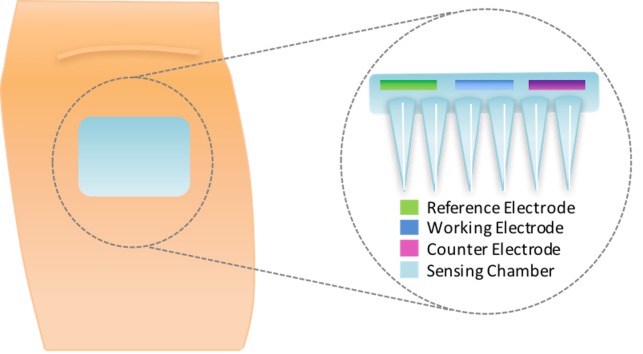
Schematic of the microneedle glucose-sensing patch on the forearm. Adapted from [62].

Microneedles and microneedle arrays have also garnered a lot of interest over recent years for interstitial fluid sensing, since this approach can offer minimally invasive methods for bio-sensing. This concept was used in the development of a glucose-sensing patch by Jina et al. [62]. The device was designed in two compartments; the first containing the microneedle array and glucose biosensor with the second containing the electronics (Figure 2). This miniaturised device spans a total area of 6 × 6 mm in which it contains 200 hollow microneedles (300 μm in length with a 50 × 50 μm lumen) [62]. Three screen-printed electrodes were used for quantifying glucose concentrations in the interstitial fluid, including a Pt-C working electrode covered with a layer of cross-linked bovine albumin serum and glucose oxidase. The sensing device was attached to the skin by an adhesive layer contouring the perimeter of the sensing pod. Detection was performed upon glucose diffusion into the microneedle array, wherein GOx could react to produce hydrogen peroxide. The production of hydrogen peroxide detected by the working electrode was proportional to the glucose concentration [62]. The electronics module of this device required the use of an external potentiostat, a microprocessor and a battery to power the device [62].

A microneedle patch platform allows the device to be in constant contact with the skin, providing permanent access to the interstitial fluid, and enabling this device to operate continuously [16]. In this particular case, the short length of the microneedles means that penetration is optimal for interstitial fluid sampling, as the microneedles do not reach the dermis layer. This minimises any damage to blood-capillaries and nerve endings found in the dermis layer. Moreover, as the microneedles penetrate the skin, contamination by sweat is avoided [16]. Tests have shown that this device can operate successfully for up to 72 h with only a 17 min lag time caused by the passive diffusion of analytes from blood into the interstitial fluid matrix [62]. To increase the lifetime of the device, the skin healing process must be inhibited. This could potentially be achieved by designing a sensing patch with microneedles of optimal length, width, tip and pitch characteristics, and by coating the microneedles with a biocompatible material exhibiting similar mechanical properties to that of biological tissue. Currently, the device must be recalibrated daily by the finger-prick approach [62]. Potential clogging of the microneedles and the distortion of their shape upon penetration of the skin can also affect the dynamics of sampling. Despite these shortcomings, this novel device holds great potential for non-invasive continuous glucose monitor.

Russell et al. [69], were the first to introduce a tattoo sensing technology using hydrogel glucose-sensing microspheres. Zhi et al. further developed this technology by encapsulating the sensors in a thin film, which offered the advantage of fast analyte transport through the device [63]. This was achieved by fabricating microvesicles through a layer-by-layer approach, which encapsulated a fluorescent labelled protein as the glucose receptor. When glucose bound to the protein, a conformational change was induced in the protein, which increased the polarity of the sensing environment. As a result, the fluorescence became increasingly quenched as the concentration of glucose increased [63]. These microvesicles were then implanted in to the dermis layer of the skin in a mouse ear, and the fluorescence lifetime was measured for glucose concentrations in a range of 1–100 mM. An advantage of measuring fluorescence lifetime meant that important issues such as light scattering and photobleaching could be avoided. This approach could also be designed for pancreatic islet transplants, which are a known treatment procedure for Type 1 diabetes patients. It is commonly seen that these cells are subject to early destruction or rejection from the innate host immune systems upon transplantation. To overcome this challenge, a biocompatible encapsulation method using fluorophores with excitation wavelengths in the NIR region holds the potential for improving the efficacy of this transplantation approach. As cellular tissues are transparent to NIR light, measurements taken via the surface of the skin could facilitate continuous non-invasive sensing [63,70]. Other methods for interstitial fluid sensing which are currently under development include sensors based on impedance spectroscopy (Pendra, by Pendragon Medical Ltd., Zurich, Switzerland) and optical transducers (C8 MediSensors Optical Glucose Monitor™ System, by C8 MediSensors, Inc., San Jose, CA, USA).

#### 2.2.2. Urine

Since 1841, urine has been used as a diagnostic fluid for diabetes [71]. It has been extensively studied, as it can be easily and non-invasively collected [14,18]. Urine is composed of metabolites, such as glucose, proteins and nitrates, as well as other dissolved salts, such as sodium and potassium. As a result, the pH of urine fluctuates between acidic pH 4.5 to basic pH 8 [14]. Due to the intermittent nature of this fluid, where collection is required for sampling, it cannot be incorporated into a continuous glucose-monitoring device [72]. Glucose can be found in urine when it is excreted from blood in elevated levels and as a result, this fluid has been investigated for the diagnosis of diabetes. A positive result occurs when the glucose levels in urine are in the 2.78–5.55 mM range [18].

#### 2.2.3. Sweat

Sweat is one of the most accessible body fluids, where its primary biological role is for thermoregulation [73]. Conveniently, for sampling purposes, eccrine glands that excrete sweat can be found all over the body, where they are particularly concentrated in multiple locations, for example in the hands, feet, lower back and underarm [56]. Sweat has been exploited for diagnostic purposes, in particular for the detection of disease markers such as sodium, potassium, calcium, phosphate and glucose [16]. It is also known that small-molecule drugs and their metabolites are present in sweat, thereby allowing the evaluation of drug efficacy [74]. Sweat can be continuously accessed and its production can be stimulated on-demand at certain locations, for example by iontophoresis [56]. By placing sensors in close contact with the skin, sweat samples can be processed rapidly without contamination [56]. For many years, sweat has been used as a sampling medium of interest in sensing devices for confirming diseases, such as cystic fibrosis and for gaining other valuable information, relating to electrolyte balance, diet, injury, stress, medications and hydration. The hydration status of individuals has become a relatively new area of interest for monitoring human performance, resulting in an increase in wearable smart devices on the global market [16,75]. Most analytes contained in sweat tend to vary significantly between basal and exercising states, as well as between individuals. The reported glucose level in sweat for healthy patients is between 0.06 and 0.11 mM and between 0.01 and 1 mM for diabetics [55]. The fluctuations in analyte concentrations result in a broad pH range of sweat, typically between pH 4.0–6.8 during exercise [76,77], which can impact on the effectiveness of chemical-sensing or biosensing techniques chosen for disease diagnosis or monitoring [14].

**Figure 3:**
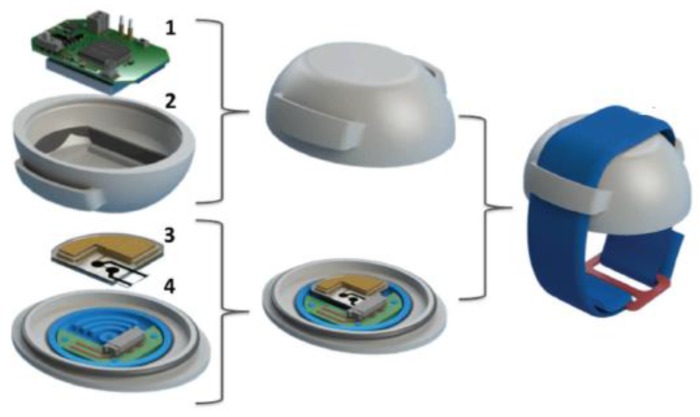
SwEatch: watch-sensing platform for sodium analysis in sweat. 1. Electronics. 2. 3D printed casing 3. Microfluidic chip and ion-selective/ion-specific electrode 4. 3D printed sweat harvester and sensor connections. Reproduced from [73], Copyright (2016), with permission from John Wiley and Sons.

An example of a device developed for sweat sensing is SwEatch by Diamond and co-workers [73]. This device was designed for sodium analysis in sweat and was fabricated using 3D-printing methods for the sensor casing and sensor connections (Figure 3). The device was powered by a lithium battery that allowed the device to function continuously for up to 3 h [73]. This approach could be easily adapted for glucose sensing in sweat by introducing a glucose sensor into the platform.

In 2010, Heikenfeld et al. began fabricating sweat-sensing patches that could stimulate sweat production, measure analyte concentrations in sweat wirelessly, and transmit that information to a smartphone [75]. This research aimed to determine physical fatigue in athletes by measuring their dehydration status using their sweat. Dehydration is known to be a reoccurring problem for professional athletes and by alerting an athlete of their oncoming over-exertion, instances such as cramping and electrolyte imbalance could be avoided, while simultaneously prompting the uptake of fluids [75]. Heikenfeld has written many reviews in this area, and his outlook for the future suggests that technology is advancing towards glucose sensing in sweat [56,74,75].

**Figure 4:**
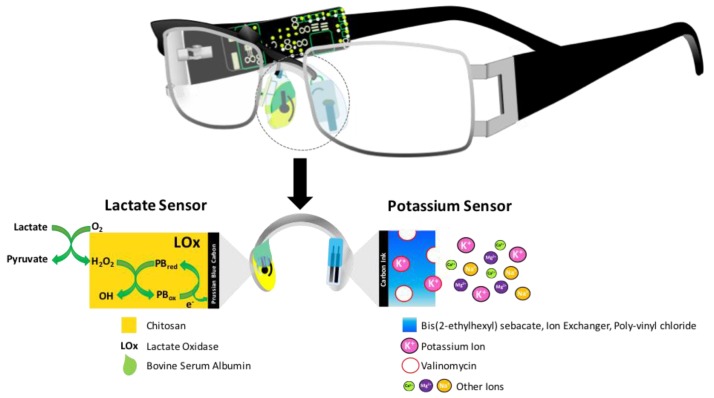
Schematic representation of the eyeglasses biosensor system, which integrates a wireless circuit board along the arms of the spectacles and two electrochemical sensors for lactate and potassium on to the nose-bridge pads. A schematic of the lactate sensor (**left**) and potassium sensor (**right**), along with the corresponding recognition and transduction events is also shown. Adapted from [80].

Wang et al. [78,79,80] have also recently been working towards the development of a continuous and non-invasive sensing device for detecting specific analytes in sweat using electrochemical sensing. In their approach, they have investigated a range of innovative sensing platforms, including wristbands [78], stick-on flexible sensors [79] and traditional eyeglasses (Figure 4) [80]. By adapting spectacles, Wang et al. have created a device that can be easily integrated into an individual’s lifestyle. This device contains an amperometric lactate biosensor connected on to one of the nose-bridge pads and a potentiometric potassium ion-selective electrode on to the other (Figure 4) [80]. Both sensors were interfaced with an electronic backbone on the glasses’ arms. The device could successfully sense lactate and potassium ions in sweat for a few hours continuously. The positioning of the sensors on separate nose pads also minimised cross-talk and facilitated separate fabrication and replacement. These instrumented eyeglasses were coupled by Bluetooth wireless data to a remote mobile host device for data analysis and visualisation. In this approach, the nose-bridge pads were designed so that the pads could be interchanged with others for multifunctional sensing applications [80]. Wang and co-workers also demonstrated that by interchanging the lactate sensing pad for a glucose sensitive one, the spectacles could be used to monitor glucose concentrations in human sweat. The results showed good blood-sweat glucose correlations in healthy individuals when the blood levels were compared with a commercially available blood-glucose meter [80].

**Figure 5:**
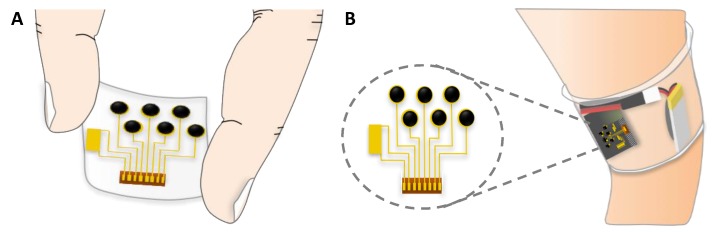
A. Flexible glucose sensor. B. Glucose sensor integrated in to a wearable wristband for non-invasive sensing in sweat. Adapted from [81].

Gao et al. [81] recently reported a non-invasive and continuous wearable glucose-sweat sensing device (Figure 5). Sensors integrated in to this Bluetooth-enabled wristband detect skin temperature, sodium, potassium, lactate and glucose concentrations in sweat [81]. An advantage of this approach is the use of multiple sensors which overcomes limitations of single, stand-alone sensors [74,81]. Due to the complex nature of sweat, multiple sensors are required to provide a more comprehensive profile of sweat composition and enable data cross-comparisons. For example, it is known that the potassium concentration in sweat is quite stable during basal and exercising states. As a result, potassium levels can be used as a reference for comparing the fluctuating concentrations of other analytes, such as glucose and enable real fluctuations to be distinguished from artefacts [81]. This device was designed to exhibit similar form factors of existing devices, such as the fitness wristbands by Fitbit Inc. (San Francisco, CA, USA), thereby encouraging user uptake to create a pathway to commercialisation. The sensors were tested individually in situ and collectively in the device. The device analyte readings showed good correlations to the normal concentration ranges in sweat during exercise and the sensors could be used for continuous operation for up to 2 h before the glucose and lactate sensing units were interchanged for fresh sensor arrays. A minimum of 10 μL of sweat was required before any sweat analysis could be achieved [81]. The sensors were placed close to the skin, to allow for immediate analysis of sweat as it emerged. Sweat was absorbed into a water-absorbent thin rayon pad for stable and reliable glucose readings, placed between the electrode sensors and skin. This flexible wearable sensing system is a promising platform for tracking multiple physiological analytes during exercise.

Rogers and co-workers [82], have examined a range of porous materials that can be used for optimising epidermal characterisation of sweat with the aim of developing thin (1 mm thick), wireless, stretchable sensors for continuous monitoring. Some of the soft materials investigated include recycled cellulose sponge, polyurethane sponge, polyvinyl alcohol sponge, cellulose paper and silicone sponge. These materials were chosen for their ability to absorb and retain sweat by capillary forces when in contact with the skin, in order to optimise the handling and analysis of the biofluid by eliminating the need for complex microfluidic systems [82]. The substrate is skin-like in nature, which makes it conformable and comfortable to wear during monitoring periods. It also is simple to integrate with a range of electronic sensors and communication platforms, such as a smart phone for simple colorimetric measurements [82]. Moreover, by employing soft skin-like polymeric materials, irritation during long-term monitoring sessions could be reduced as direct contact of the electrodes with the skin is prevented, by having the hydrogel sandwiched between the skin on one side and the metal electrode substrate on the other. The device developed by Rogers et al. could successfully monitor the volume and pH of sweat, as well as the physiologically relevant concentrations of copper (Cu^+^) and iron (Fe^2+^) ions (0.8–1 mg/L) for period of 2 h [82].

Rogers and co-workers [83], have also investigated soft ferromagnetic materials for skin-interfaced electrodes. This material was designed for intimate and adhesive contact with skin ensuring a device that is conformable and robust enough for continuous operation compared to conventional hydrogel-based alternatives. The device is also paired with a separate standing platform, to which the electrodes are magnetically attached to facilitate for cleaning or replacement of sensor units [83]. The main advantages of this approach included the conformability and resilience of the sensors to allow for natural movements on body during sensing periods and direct integration of the wireless electronics for data collection and communication. Moreover, the sensors minimised noise from motion artefacts by incorporating a magnetic bi-layer material [83]. In comparison to conventional hydrogels, which are known to shrink or swell in response to certain analytes, this magnetic material retained its shape when exposed to biofluids or air. Continuous operation of the device was demonstrated for a period of two days (50 h) [83]. Measurements performed included electroencephalograms (EEGs) to monitor electrical activity of the brain [84], electromyograms (EMGs) to detect electrical activity in muscles [85], electrocardiograms (ECGs) [86] and electrooculography (EOG) [87]. The designs are compatible with the most sophisticated electrode potential monitoring systems that are currently commercially available. The authors also suggest that the material can be readily adapted for detecting other epidermal analytes of interest [83].

Overall, although sweat sensing for diagnostic data is very promising, there are also some concerns associated with this sensing fluid [56]. The main challenges include (1) limited fundamental knowledge about the sensing fluid, compared to other physiological fluids such as blood which are much better understood; (2) sampling issues associated with sweat production by exercising, iontophoretic stimulation; heat or carbon dioxide [76] (3) the skin surface can also act as a contaminant; as sweat traveling across the skin can mix with fresh emerging sweat and contaminate the sample (4) the pH range of sweat fluctuates over a wide range between pH 4.0–6.8 which can interfere with some sensing approaches [77] and (5) the rate of sweat production is variable and can be quite low (1 nL/min/mm^2^) [56]. However, this low sweat rate can be compensated for by increasing the area sampled for sweat analysis. Although most of these effects can be adjusted for in prototype technology innovations, some biological factors such as the variable nature of sweat pH, and variable concentrations of ions such as sodium and chloride remain a significant hurdle. Broad pH ranges can affect enzymatic sensors and influence the concentration of weak acids or weak bases in sweat, such as phosphates, chlorides or salts of organic acids like lactic acid. Therefore, these concentrations may be observed at slightly higher concentrations in comparison to other fluids, such as blood [56,88].

#### 2.2.4. Breath Analysis

Breath analysis is another means of tracking the health status of an individual [14,89]. Volatile organic compounds (VOCs) are generated as by-products from metabolic pathways within the body. These biomarkers migrate around the body via the circulatory system, pass over the alveolar interface and are exhaled in breath [90]. A molecular breath signature can be established by measuring the ratio between VOCs in breath [90,91]. This signature is composed of over 3500 VOCs, consisting of hydrocarbons, ketones, aldehydes, alcohols, esters, nitriles and aromatic compounds, whose concentrations can be affected by specific diseases [90]. For example, 112 of these VOCs have been found to be specifically related to cancer [91].

Nanomaterials can be incorporated into sensing elements for monitoring acetone concentrations in breath, as an alternative to glucose monitoring for diabetes [92]. Ethanol and methyl nitrite have also been identified as biomarkers for diabetes [90]. Acetone levels in blood are approximately 330 times higher than in breath, at 0.1–2 ppm for healthy individuals’ post-glucose loading and reaching to up to 103.7 ppm in critically ill diabetic patients [59]. Therefore, the sensing units must have a high sensitivity to detect VOCs in the nanomolar to picomolar concentration ranges, where recent examples of such devices lack specificity for concentrations of biomarkers relating solely to diabetes [59]. Jiang and co-workers have reported a breath acetone analyser, which can detect acetone levels in diabetic patients [59]. Unfortunately, the device requires a controlled external atmosphere in order to diagnosis diabetes accurately, where the reported diagnosis accuracy is estimated at 74%. However, other factors can affect acetone levels, including fasting, exercising, dieting and intra-individual variation, thereby limiting the use of acetone as a specific biomarker for diagnosing and monitoring diabetes [93].

For this technology to be fully efficient, the sensor must also integrate easily into a portable meter, and provide a means for non-invasive, inexpensive and qualitative monitoring to be implemented into a rapid response tool for early-diabetes diagnosis [89,90,91]. Although breath can be more readily evaluated in comparison to blood or urine, there are some shortcomings when attempting to design a device with fast analytical responses in breath. The analysis of VOCs in breath is more challenging due to external air interferents and the wide complex range of VOCs detectable, although more comprehensive analysis techniques can be incorporated, such as GC-MS, to provide rich-information regarding the breath composition [16,89,91,93]. However, the device currently remains expensive and non-portable, although significant advances in miniaturisation have occurred.

Electronic-nose (e-nose) technology has also been applied to continuous monitoring of breath. These portable sensors were designed to mimic olfactory sensing by breath analysis using sensor arrays that generate response patterns that can be related to the composition of breath [59]. E-noses have been primarily designed for assessing volatile mixtures of biomarkers or VOCs in breath for lung diseases. The e-nose generates response patterns when exposed to the breath samples, and these patterns are compared to database libraries of molecular breath signatures for known diseases, such as chronic obstructive pulmonary disease (COPD) [94], upper respiratory tract infections (URTI) [95] and pulmonary tuberculosis (TB) [96]. Currently these devices have found applications in the food [97], environmental and chemical industries [98], as well as in clinical trials for the recognition of lung cancer [99], asthma [100], pulmonary arterial hypertension [101] and diabetes [59]. However, a major obstacle, which must be overcome before these devices reach the consumer market is the elimination of issues involving external air contaminants. In order to standardise the sampling procedure for accurate detection, stringent control to remove any external air contaminants is necessary for background correction of the device. This is not only related to the dead-space in the device, but also the atmosphere the patient is exposed to [89]. In order to reach its full commercial potential, further development and standardisation must be implemented. Moreover, controversy still exists regarding the reliability of this approach for accurate correlation of response patterns to underlying health conditions [16,89].

#### 2.2.5. Saliva

Saliva is a complex fluid containing many analytes that permeate from blood, thereby in principle providing a useful insight in to a person’s emotional, hormonal, metabolic and nutritional state [15,102]. As a result, saliva has been investigated as an alternative fluid for non-invasive glucose sensing, with glucose levels for a healthy individual ranging from 0.23 to 0.38 mM and between 0.55 and 1.77 mM for diabetics [57]. Although a relationship between glucose levels in blood and saliva obviously exists, it is not well understood, and relating saliva concentration to therapeutic intervention is therefore more challenging. However, saliva offers many advantages for diagnostics, the main benefit being that saliva can be collected in a non-invasive fashion. As a result, there have been many emerging technologies reported for continuous and non-invasive glucose detection in saliva, using everyday dental platforms, including mouth guards and dentures, as well as novel devices, such as dental tattoos [15,103].

**Figure 6:**
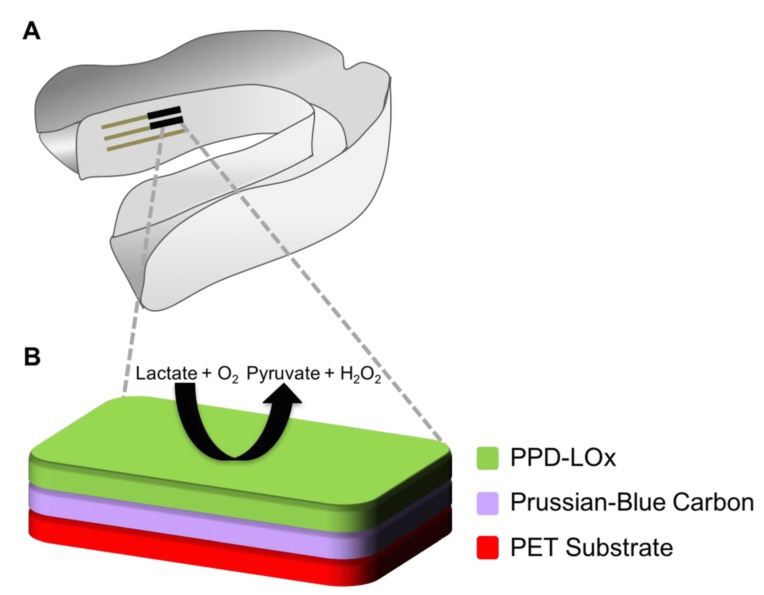
Lactate sensing mouth guard for non-invasive continuous lactate sensing in saliva. (**A**) Mouth guard sensing platform showing integrated printable 3-electrode system, with a lactase enzyme immobilised on the working electrode; (**B**) Detailed representation of the working electrode and components. Adapted from [103].

Several research groups, including that of Kim et al. [14,103], have investigated the potential use of a mouth guard as a minimally invasive continuous monitoring platform for metabolite sensing in saliva. This sensing platform encompasses a printable enzymatic electrode, based on lactate oxidase, for the detection of salivary lactate (Figure 6) with high sensitivity, selectivity and stability in human saliva samples [103]. This amperometric electrochemical sensing approach uses a poly-orthophenylenediamine (PPD)/lactate oxidase reagent layer with a printable Prussian-blue transducer, where the Prussian-blue reagent acts as the ‘artificial peroxidase’ to offer a highly selective detection approach for hydrogen peroxide in the catalytic reaction [103]. The Prussian-blue reagent was previously used for oral treatment of heavy metal poisoning, such as thallium and caesium, for which it is widely accepted as safe under physiological conditions and at high oral dosages. PPD has also been commonly used for electropolymeric entrapment of oxidases, and its relatively low oxidation potential in comparison to other electroactive species, such as ascorbic acid or urea, facilitates protection of the biosensor surface, and enhances stability of the sensing components [103]. This sensing approach also has the potential to be used for glucose biosensing, by replacing the lactate sensor with a glucose sensor, as the sensing principles of these electrochemical biosensors and their associated electronics are very similar.

**Figure 7:**
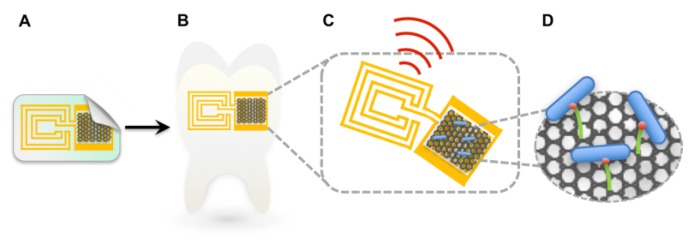
Glucose-sensing tattoo printed on to a tooth platform as a non-invasive continuous monitoring device. (**A**) Graphene printed on bio-resorbable silk with contacts containing a wireless coil; (**B**) Bio-transfer of sensor on to tooth enamel; (**C**) Magnified image of the sensing unit with a wireless readout system; (**D**) Self-assembly of pathogenic bacteria bound by peptides on nanotransducer surface. Adapted from [104].

Intraoral dental accessories have also been of interest for non-invasive and continuous monitoring to provide information regarding a patient’s health status. A tooth has the potential to act as a continuous monitoring device as it is in constant contact with the patient’s saliva. Mannoor et al. [104] have developed a bacterial detection approach whereby a graphene-based nanosensor was printed on to water-soluble silk and transferred on to tooth enamel (Figure 7). This sensing tooth incorporates a resonant coil to prevent the need for a power source and external connections. The device operates by the self-assembly of antimicrobial peptides on to the single sheet of graphene, where the bio-selective analysis of bacteria can be performed at a single cellular level. Preliminary results showed great specificity, response time and single-molecule detection abilities for this sensor, however this sensing application must still be tested on-body for real-time analysis [104]. This approach could potentially be adapted for detection of other analytes such as glucose, by means of a chemical glucose sensor immobilised on to a water-soluble silk layer attached to the tooth enamel.

Zhang and co-workers [71] have fabricated a disposable microfluidic device that has been developed as a diagnostic tool for quantifying saliva-glucose concentrations by electrochemical methods. The glucose-sensing chip houses a working electrode, a counter electrode and reference electrode [71]. The intricate device design consisted of a working electrode functionalised with single-walled carbon nanotubes, to promote electron transfer for the glucose oxidase reaction. Gold nanoparticles were incorporated to enhance this signal sensitivity, as well as to promote increased enzyme attachment. Chitosan, a non-toxic biocompatible polysaccharide, was also employed for promoting film formation and adhesion characteristics in order to enhance glucose oxidase immobilisation [71]. This device showed impressive linearity for glucose detection in the range of 0.017–0.8 mM [71], correlating to saliva-glucose levels in healthy patients.

#### 2.2.6. Ocular Fluid

The fluid surrounding the eye and ocular tissue, also known as the aqueous humour, contains many analytes present in blood. This complex fluid can be excreted from the body as an extracellular fluid in the form of tears. Analytes found in this fluid, such as glucose, ascorbic acid, lactate, proteins, peptides, hormones, carbohydrates, electrolytes, lipids and chloride, can offer great insight into an individual’s health status [15,105]. As a result, this fluid has been investigated for non-invasive and continuous glucose monitoring.

**Figure 8:**
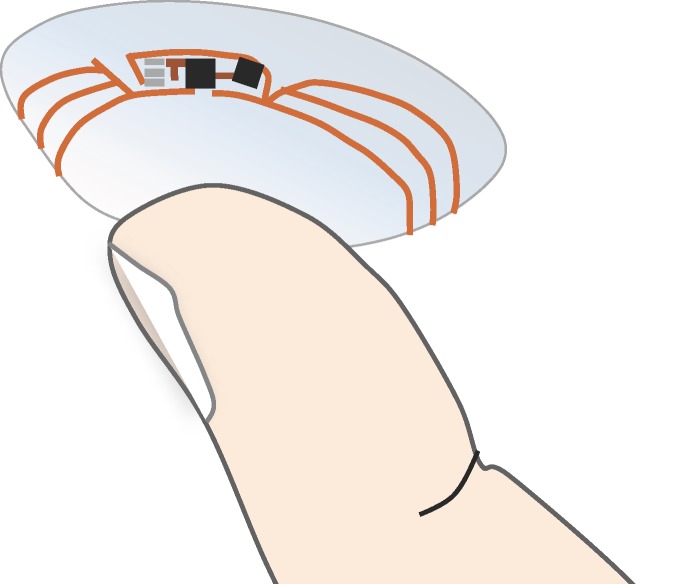
Google and Novartis smart-contact lens. Adapted from [107].

Recently, the Google[X] lab (Mountain View, CA, USA) and Novartis (Basel, Switzerland) have collaborated on the development of glucose sensing technologies in the aqueous humour. The Google[X] lab was founded to “find new solutions to big global problems” with diabetes falling into this category [106]. In 2014, they announced their goal to create a smart-contact lens, which they hoped would overcome glucose-monitoring obstacles associated with current methods, which are either invasive, in the case of implanted wearable devices or non-continuous, in the case of the finger-pricking approach. This novel technology incorporates an electrochemical battery-operated enzymatic glucose sensor, utilising the enzyme glucose oxidase (GOx), in a microchip sandwiched between two layers of a soft contact lens, as represented in Figure 8 [106,107]. A tiny sensor relays data to a mobile device, from which the patient or medical practitioner can read the corresponding glucose levels in the ocular fluid [108,109,110]. This redox reaction is monitored through the production of hydrogen peroxide at the working electrode to quantify the glucose concentration in the ocular fluid [111]. However, there are disadvantages to an electrochemical sensing approach, which can be related to the use of enzymes leading to the production of corrosive hydrogen peroxide as a by-product or interference from electroactive species in the ocular fluid, such as ascorbic acid, lactate or urea [105,109]. Blinking may also stimulate a movement artefact in the wireless sensor signal. In addition to the battery source embedded in the lens, an external power source must be provided for enabling efficient sensor function and to facilitate wireless communication of the data. This is a very active area of research, with multiple approaches under investigation into the production of a safe biocompatible battery powered device [112]. These include additional power sources external to the device that would wirelessly transmit power, for example through radio frequency induction, using solar cells to harvest energy, or using biofuel cells. Biofuel cells could be used in a second wearable device to supply chemical energy, which would be converted to electrical energy, to power the biosensor. This separate power source would need to be within close proximity of the eye in order for the smart-contact lens to function efficiently [112].

Using a contact lens as a sensing platform does hold many advantages, including real-time continuous and non-invasive glucose monitoring, as the lens would be in constant contact with the aqueous ocular fluid [20,105,113]. Disposable contact lenses are typically replaced every 24 h, which is a reasonable period for reliable continuous biosensor operation. Blinking and tear secretion also allow for natural, fresh sample replenishment for accurate glucose concentration measurements throughout the day [105]. By incorporating a glucose sensor in to a commercially available lens, not only could this lens provide a form of corrective vision but a monitoring function too [20,105,110]. It is well known that critical side effects associated with diabetes are eye damage or blindness [20], due to glycation of proteins in the blood vessels of the eye [37]. To account for such ocular deteriorations a detection function, potentially based on imaging, for glycation of vascularised tissues could later be incorporated in to the lens.

Other research groups, such as Yao et al. have worked with Google, to investigate the use of contact lenses as a biosensing platform for quantifying glucose concentrations in the ocular humour. GOx was the enzyme employed in an amperometric glucose sensor for this purpose. The sensor was immobilised as a screen-printed structure on to a polymeric substrate in the shape of a lens [72]. An amperometric sensing method was chosen since the oxidation and reduction potentials of this reaction can be directly associated with a measurable current signal, which is proportional to the concentration of the analyte being quantified [105]. The preliminary results showed some promise, with the lens reported to sense glucose in the range of 0.1–0.6 mM [72]. This correlates with ocular glucose levels of 0.05–0.5 mM for healthy individuals, increasing to up to 5 mM for critically ill diabetics [20]. The ocular glucose levels in comparison to blood-glucose levels are approximately 10 times smaller with a lag time of about 10 min [14,105,114]. Although this smart lens showed rapid response times of up to 20 s and good reproducibility [72], it did not manage to demonstrate non-invasive and continuous glucose-monitoring. Many issues associated with tear fluid interference and challenges related to powering the device are still under development [105]. Kagie et al. [115] and Iguchi et al. [114] proposed an alternatively designed sensor, using the same glucose sensing methodology, which was inserted into the tear canal. Kagie et al. [115] showed that the sensor could be fabricated by screen-printing approaches and Iguchi et al. [114] demonstrated the sensor production by microfabrication techniques. The sensors fabricated by both approaches were reported to have high selectivity for ocular-glucose, but further tests are required to determine the effects of interfering electroactive species such as ascorbic acid, lactate or urea, all of which are present in the aqueous humour [114,115].

Recently, Badugu et al. introduced an optical chemical sensor for glucose detection in the ocular fluid [20]. In this case, they used boronic acids (BAs) attached to a fluorescent component to directly sense glucose by optical means [20,21,36,37,38,39,45,116,117]. On interaction of the fluorescent BA sensors with increasing saccharide concentrations, a decrease in the fluorescence intensity of the BA sensors occurred. For example, when using a fluorescent quinoline moiety a decrease of ~30% was observed upon introduction of 100 mM glucose [20,21]. This sensing mechanism was attributed to fluorescence quenching via a charge neutralisation-stabilisation mechanism. The anionic boronate ester formed on glucose binding was stabilised by a positively charged N atom in the quinoline structure, which consequently quenched the fluorescence of the BA sensor [20,21]. The main advantage for using chemical sensors over enzymatic sensors is that the sensing detection and communication mechanisms do not require any power. Chemical sensing by optical means, eliminates the need for external sources of energy to power the device when the material is self-reporting. The concentration changes detected by the sensor induce an optical change in the material, which are used to communicate the analytical response. As a result, by using a chemical sensing approach the sensing components can be greatly simplified. Moreover, the production of corrosive hydrogen peroxide is eliminated, overcoming a significant disadvantage of the electrochemical sensing approach [20].

Other groups working towards non-invasive monitoring of glucose in the ocular fluid include Jeong, et al. and Microsoft Inc. (Redmond, WA, USA) in collaboration with Evans and co-workers. Jeong, et al. [118] have developed Raman sensitive, silver plasmonic, nanostructures that are responsive to glucose. These nanostructures used solvent-assisted nanotransfer printing to attach the plasmonic glucose sensing material on to a commercially available contact lens. Nanotransfer printing in this way created narrow passageways or ‘hot spots’ in the nanostructure, to take advantage of the surface-enhanced Ramen scattering in the sensing material to detect small molecules, such as glucose, non-invasively in the ocular fluid. The approach is clever in that it uses retina-safe laser excitation to measure glucose concentrations within the range of 0.1–10 mM [118].

Microsoft, in collaboration with Drew Evans’ group from the University of South Australia (Mawson Lakes, SA, Australia), have designed hydrophilic organic electrodes that could be incorporated in to flexible hydrogels, such as a contact lens [119]. In their approach, they have fabricated polymeric coatings that were engineered to be biocompatible and conducting, which could be grown directly on to a contact lens. Evans and co-workers, deposited the conductive polymer poly(3,4-ethylene-dioxythiophene (PEDOT) on to the lens using oxygen plasma techniques. This lens was designed to advance the development of silicone hydrogels with technologically relevant properties, such as conductivity, to pave the wave for conductive hydrogel electrodes [119]. Ultimately, this lens would self-report relating optical changes to glucose concentrations. An advantage of this passive device is that no batteries or wireless connections are required to receive and transmit crucial information. Currently, work is underway in the group to test the robustness of the coatings, to further develop the lens towards disease diagnosis and monitoring, and other alternatively interesting applications for military camouflage apparel, where electricity would induce a reversible colour change in the polymer from brown to green [120].

## 3. Conclusions

Wearable sensors have the potential to play a major role in the continuous and non-invasive monitoring of biomarkers for diabetes and other disease conditions. The majority of sensors described in this review still require further clinical evaluation before they can be approved for medical use. The interest of companies such as Google, Novartis and Microsoft suggests there is really significant market potential for new approaches to self-monitoring and diagnosing. A key enabling step will be to create a clearer understanding of the relationship between the diagnostically relevant concentrations of key disease markers in blood compared to other physiological fluids. Existing wearable devices such as fitness bands and smartwatches, which already dominate the consumer technology market, provide an information base that can be expanded to encompass disease monitoring or diagnosis, and provide a tangible impact on health and wellness. However, unless these wearable technologies can provide additional insights into the wearer’s health, be integrated in to clinical practices and promote actionable behavioural change, their positive impact may continue to be limited.

## Figures and Tables

**Table 1:** Summary of glucose concentrations postprandial and pH values measured in physiological fluids of healthy and diabetic patients.

Physiological Fluid	Biomarker	Concentration for Healthy Patients’	Concentration for Diabetic Patients’	pH
Blood	Glucose	4.9–6.9 mM [19]	2–40 mM [20,40]	7.35–7.45 [14]
Interstitial Fluid	Glucose	3.9–6.6 [53]	1.99–22.2 [54]	7.2–7.4 [14]
Urine	Glucose	2.78–5.55 mM [18]	>5.55 mM [18]	4.5–8 [14]
Sweat	Glucose	0.06–0.11 mM [55]	0.01–1 mM [55]	4.5–7 [56]
Saliva	Glucose	0.23–0.38 mM [57]	0.55–1.77 mM [57]	6.2–7.6 [58]
Ocular Fluid	Glucose	0.05–0.5 mM [21]	0.5–5 mM [20,21]	6.5–7.6 [18]
Breath	Acetone	0.1–2 ppm [59]	0.1–103.7 ppm [59]	7.4–8.1 [60]

**Table 2:** Summary definitions of specialised approaches for glucose sensing in interstitial fluid.

Sensing Technique	Explanation	Reference
Reverse Iontophoresis	Reverse iontophoresis is the passing of a current over the skin to drive ions from the interstitial fluid and onto the surface of the skin, where they can be analysed. This results due to the increased negative charge across the skin which drives cations from the body on to the skin surface.	[65]
Thermal Emission	Thermal emission, infrared thermography, infrared imaging or thermal imaging is a non-contact tool that uses the surface body temperature to gain insight in to the detection of diseases, flow of blood or muscular performance of an individual.	[66]
Photoacoustic Detection	Photoacoustic spectroscopy uses a form of light absorption to detect an analyte concentration in a biofluid, such as blood. Light from a laser source is absorbed by blood which generates heat in a localised region. The heat induces ultrasonic pressure waves in blood, which can be detected by an ultrasonic transducer and directly related to an analyte concentration that absorbs at the laser wavelength.	[10]
Sonophoresis	Transdermal delivery of drugs by ultrasound.	[67]
